# GENDER-AFFIRMING SCROTECTOMY: INITIAL DESCRIPTION AND OUTCOMES

**DOI:** 10.51894/001c.122878

**Published:** 2024-08-30

**Authors:** Kitan Zoltin, Briar Shannon, Ryan Timar, Nabeel Shakir

**Affiliations:** 1 College of Osteopathic Medicine Michigan State University

## 28

### INTRODUCTION

Patients may seek gender-affirming orchiectomy and scrotectomy (GAOS) to alleviate dysphoria related to the scrotum and testes, while allowing for penetrative intercourse, if desired.

### OBJECTIVES

Techniques and outcomes of scrotectomy in this specific setting have not been described to date. Here we discuss our initial experience with GAOS as an option for patients who do not necessarily desire other gender-affirming genital surgeries.

### METHODS

All patients who had undergone GAOS procedures from 2021 to 2022 at our institution were reviewed. The World Professional Association for Transgender Health (WPATH) criteria for surgical treatment were met for all patients preoperatively. In addition, patients expressed understanding that vaginoplasty would not be recommended after scrotectomy. Patients were offered a choice of two approaches depending on their surgical goals: excision of a majority of scrotal tissue and primary closure of the perineal wound, or excision of all rugated skin with mons and groin (Y-flap) advancement. Preoperative demographic data, baseline sexual function, intraoperative findings, and postoperative outcomes were collected. Patient-reported outcomes (PROs) derived from the PROMIS^®^ Sexual Function questionnaires were sent to all patients postoperatively and collected.

### RESULTS

The median operative time was 152 min. All patients were discharged home the same day. All five patients had procedures complicated by the presence of a scrotal web, and two experienced postoperative complications ranging from mild tethering with erections to wound dehiscence requiring skin graft. At median follow-up (97 days), all reported satisfaction with outcomes. Patients who experienced postoperative complications reported no change to their libido and the ability to achieve erection and orgasm. Further patient follow-up is being collected.

### CONCLUSIONS

GAOS is a well-tolerated procedure that can reasonably address dysphoria related to the scrotum and testes. It is associated with high satisfaction and a low risk of postoperative complications causing long-term sequelae. Further and long-term study of this emerging procedure is warranted.

